# miR-30c affects the pathogenesis of ulcerative colitis by regulating target gene VIP

**DOI:** 10.1038/s41598-024-54092-y

**Published:** 2024-02-12

**Authors:** Xiang Dong, Yuling Zhan, Minghui Yang, Suwan Li, Hailun Zheng, Yu Gao

**Affiliations:** 1 School of Life Science, Laboratory Animal Center, Bengbu Medical College, No. 2600 Donghai Road, Bengbu, 233030 China; 2 Bengbu Medical College Key Laboratory of Cancer Research and Clinical Laboratory Diagnosis, Bengbu Medical College, Bengbu, China; 3 School of Basic Courses, Bengbu Medical College, Bengbu, China; 4 Department of Gastroenterology, The First Affiliated Hospital of Bengbu Medical College, Bengbu, China; 5 Anhui Province Key Laboratory of Translational Cancer Research, Bengbu Medical College, Bengbu, China; 6 Laboratory Animal Center, Bengbu Medical College, Bengbu, China

**Keywords:** Molecular biology, Diseases, Gastroenterology

## Abstract

MicroRNAs play a crucial role in regulating the epithelial barrier and immune response, which are implicated in the pathogenesis of ulcerative colitis (UC). This study aimed to investigate the role and molecular mechanism of miR-30c in the pathogenesis of UC using a dextran sulfate sodium salt (DSS)-induced colitis model, which is similar to ulcerative colitis. Wild-type (WT) and miR-30c knockout (KO) mice were assigned to either control or DSS-treated groups to evaluate the influence of aberrant miR-30c expression on UC pathogenesis. The disease activity index, inflammatory factors, and the extent of pathological and histological damage in colon tissues were analyzed. The effect of miR-30c on vasoactive intestinal peptide (VIP) gene expression was validated through luciferase reporter assay, qRT-PCR, Western blotting, and immunohistochemistry. The results showed that miR-30c KO mice with DSS-induced colitis model showed more severe phenotypes: significantly higher disease activity indices, significant body weight loss, reduced length of the colon of mice, increased number of aberrant crypt structures, reduced mucus secretion, and significant differences in inflammatory factors. These findings suggested that the absence of miR-30c might promote DSS-induced colitis, and the targe-regulatory effect of miR-30c on VIP might play an important role in the development of colitis.

## Introduction

Maintaining good bowel function is crucial for overall human health. Unfortunately, inflammatory bowel disease (IBD) is a lifelong inflammatory disease affecting the gastrointestinal tract, causing diarrhea, urgency, and abdominal cramping^1^.Ulcerative colitis (UC), one of the two main forms of IBD, is predicted to have a worldwide incidence of up to 5 million cases by 2023^2^. It can be diagnosed at any age and involves inflammation of both the rectum and the colon^3^. The relapsing–remitting disease characteristic of UC requires different therapeutic approaches to induce and maintain remission^4^, but only 10–15% of patients achieve remission with current induction therapies^5^. To expand the therapeutic options for UC, different types of biologics and oral small molecules are continuously being developed^6^. More objective biomarkers can be used not only to stratify the risk of clinical features and thus define the overall disease severity, but also provide ideas for developing drug therapy.

To the best of our knowledge, the development of UC is linked to the interaction of epithelial barrier defects, dysregulated immune response, and flora imbalance in the gut^2^. Patients with UC are known to have a significantly higher risk of developing colorectal cancer as compared to the general population, and the risk increases progressively with the duration and severity of the disease^7^. It has been reported that microRNA molecules are effective in improving intestinal inflammation by influencing immune-targeted signaling pathways, regulating intestinal epithelial proliferation and differentiation, and ameliorating intestinal inflammatory states. They are involved in impacting the body’s immune cells, immune responses, and defense against pathogenic microorganisms^8^. For example, the overexpression of miR-155 in intestinal mucosal tissues was positively correlated with the development of UC, which might promote epithelial-mesenchymal transition and accelerate the progression of UC through the activation of the Wnt signaling pathway and the down-regulation of E-calmodulin expression^9^. Recent studies have shown that microRNA-30c (miR-30c), which regulates inflammation, immunity, and tumor growth, is aberrantly expressed in colon cancer tissues and is significantly associated with cancer progression and clinical prognosis^10^.

Vasoactive intestinal peptide (VIP) is a polypeptide hormone comprising 28 amino acids, which are expressed in the brain, heart, lungs, kidneys, bladder, pancreas, and gastrointestinal tract^11^. Initially isolated from the gut, VIP was identified as a vasodilating peptide that alters intestinal blood flow^12,13^. It is one of the most abundant neuropeptides in the human body and is secreted mainly by neuronal tissues^14^. Immune cells such as eosinophils, mast cells, and lymphocytes also can secrete VIP^15^, which regulates the homeostasis of the immune system as a cytokine or chemokine^16^. VIP exhibits anti-inflammatory effects on immune cells in inflammatory diseases, including IBD^17,18^. It has been reported that VIP attenuates colitis-associated inflammation and diarrhea after systemic administration, making it a promising therapeutic option for IBD management^19^.

Currently, there is a lack of clear findings on the role of miR-30c in the pathogenesis of ulcerative colitis, its corresponding downstream target genes, and the signaling pathways.This study aimed to explore the role of miR-30c on UC pathogenesis in the mouse and human. To investigate the effect of miR-30c on UC in mice, a colitis model was established using dextran sulfate sodium salt (DSS) induced. The study also explored the targeting relationship between miR-30c and VIP, and its potential impact on UC and DSS-induced colitis through VIP regulation.

## Results

### Low miR-30c expression exacerbated DSS-induced colitis in mice

**Figure 1:**
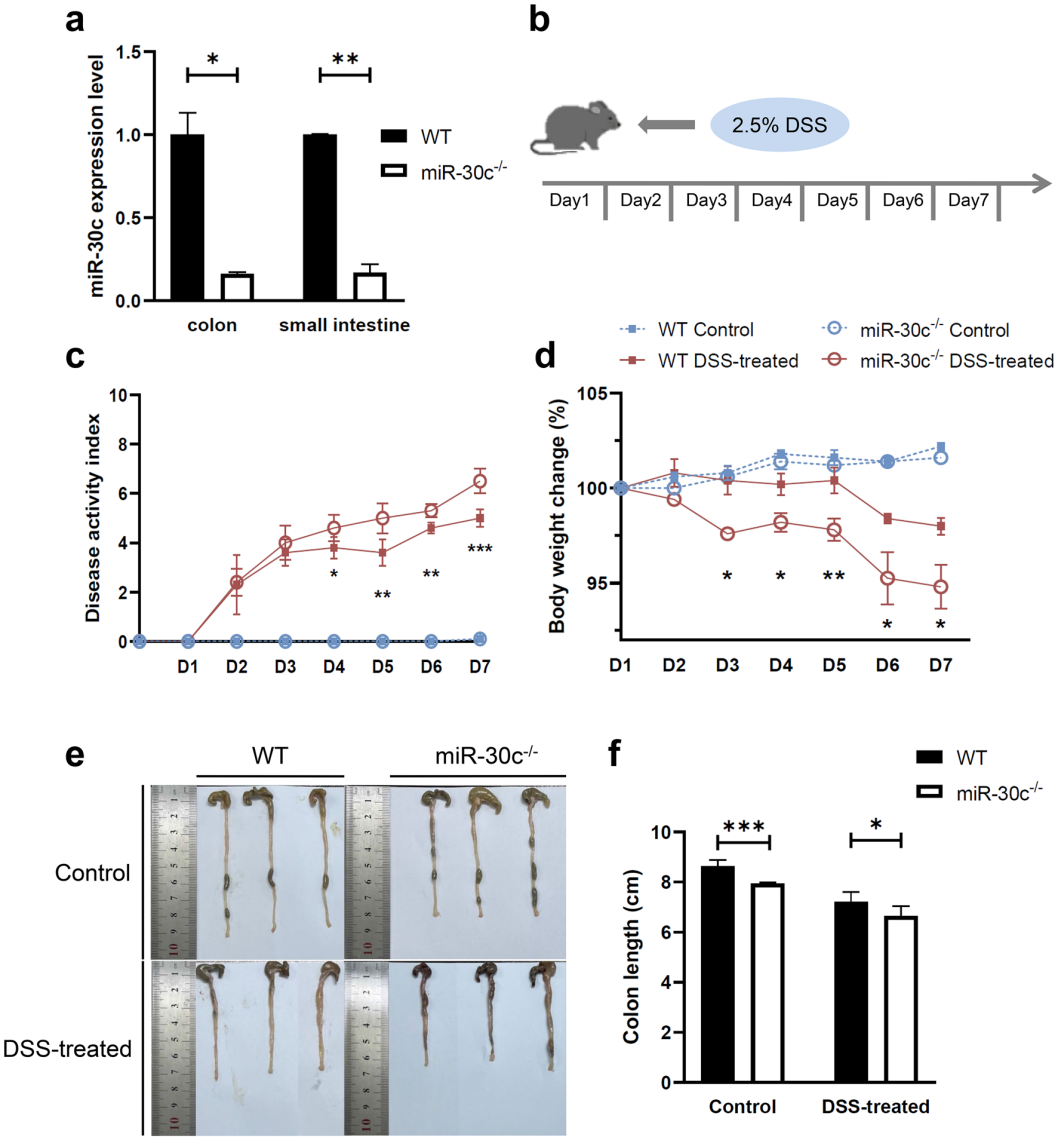
Low miR-30c expression exacerbates DSS-induced colitis in mice. (**a**) The miR-30c expression level in colon and small intestine tissues of WT and miR-30c KO mice (n = 3/group). (**b**) Diagram of colitis model in mice induced using DSS (n = 6/group). (**c**) DAI scores of colitis among WT and miR-30c KO mice in control and DSS-treated groups (WT DSS-treated group versus miR-30c^-/-^ DSS-treated group, n = 6/group). (**d**) Daily change curve of body weight of different groups’ mice (n = 6/group). (**e**) Representative photograph of different groups’ mice colons on day 8 of the experiment. (**f**) Statistical analysis of colon length in different groups’ mice (n = 6/group). Data are presented as the means ± SD and were analyzed with Student’s *t*-test (*P < 0.05; **P < 0.01; ***P < 0.001).

The expression level of miR-30c was examined in the colon tissues of mice in order to verify the construction of miR-30c knockout mice to ensure the accuracy of the subsequent experiments. The results demonstrated that the expression level of miR-30c was down-regulated (Fig. 1a). The 2.5% DSS-induced colitis model in mice was established to prove that the low expression of miR-30c promoted the pathogenesis and progression of UC (Fig. 1b). And the daily disease activity index of mice was scored according to weight loss (%), stool consistency and occult blood during DSS-induced colitis. It showed that the daily DAI (disease activity index) scoring of miR-30c KO mice after modeling was consistently higher than that of WT mice (Fig. 1c). Meanwhile, WT mice showed improvements in the severity of DSS-induced colitis, with a decrease in the disease activity index on day 5. In contrast, miR-30c KO mice had a persistently elevated disease activity index. Also the percent daily weight loss of miR-30c KO mice from the third day of modeling was significantly higher than that of WT mice (Fig. 1d). It showed congestion, edema and brittleness in DSS-treated group mice colon (Fig. 1e). And miR-30c KO mice showed significantly shorter colon length compared to WT mice (Fig. 1f).

### The inflammation in miR-30c KO mice

**Figure 2:**
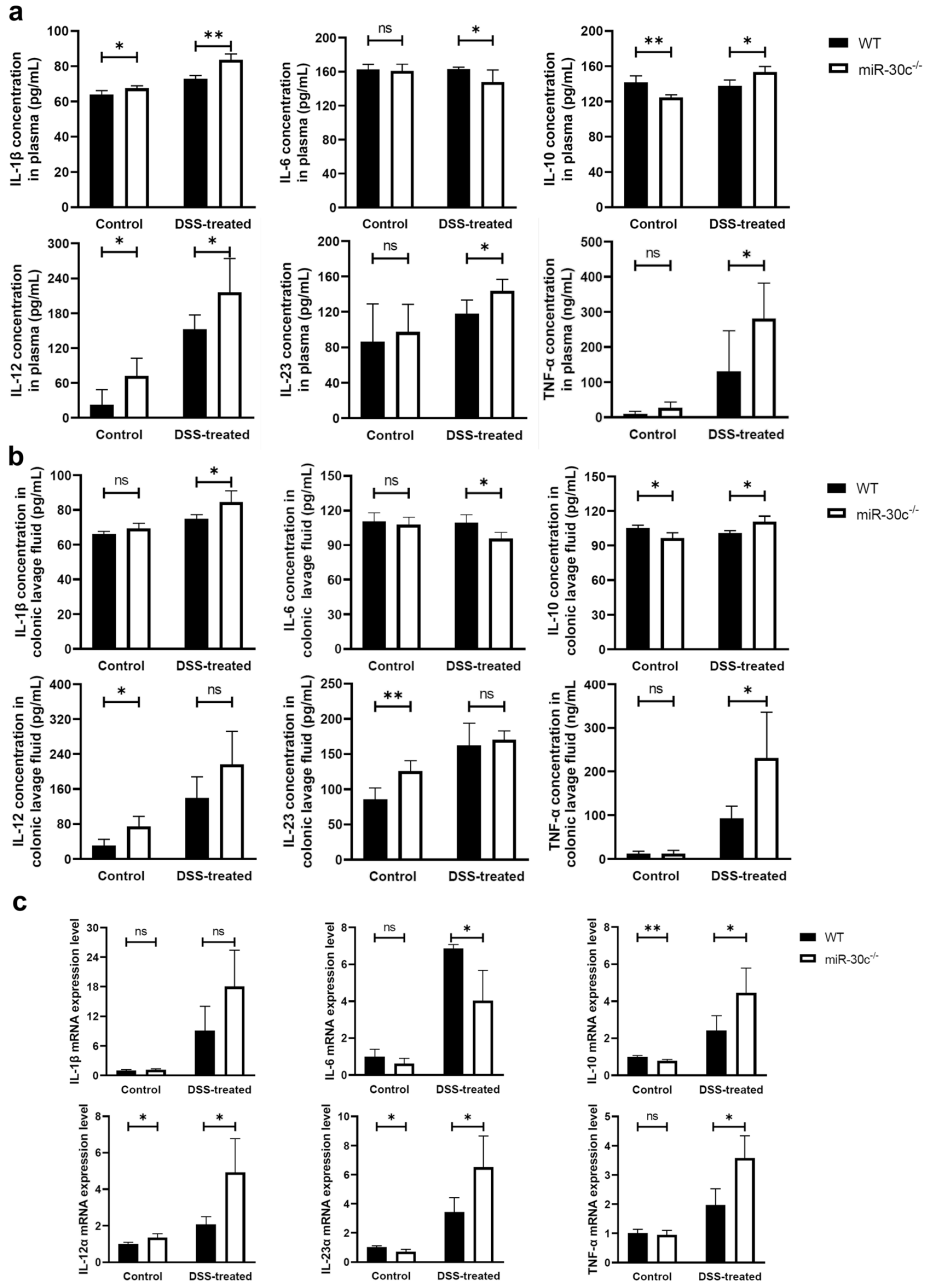
The inflammation in miR-30c KO mice IL-1β, IL-6, IL-10, IL-12, IL-23, and TNF-α. (**a**) The levels of IL-1β, IL-6, IL-10, IL-12, IL-23, and TNF-α in plasma samples detected by ELISA (n = 4–6/group). (**b**) The levels of IL-1β, IL-6, IL-10, IL-12, IL-23, and TNF-α in colonic lavage fluid samples detected by ELISA (n = 4–6/group). (**c**) The mRNA levels of IL-1β, IL-6, IL-10, IL-12, IL-23, and TNF-α in the colonic mucosa samples (n = 4–6/group). Data are presented as the means ± SD and were analyzed with Student’s *t*-test (*P < 0.05; **P < 0.01; ***P < 0.001).

The levels of IL-1β, IL-6, IL-10, IL-12, IL-23, and TNF-α in plasma of WT mice and KO mice with/without DSS were showed in Fig. 2a. The levels of these inflammatory fators in in the colonic intestinal lavage fluid were also measured. And the data were shown in Fig. 2b. In the samples of plasma and the colonic lavage fluid, the levels of IL-1β IL-12, IL-23, and TNF-α in miR-30c KO mice were significantly higher than those in WT mice with DSS treated. Thelevels of IL-10 in miR-30c KO mice were significantly higher than those in WT mice, but it showed the opposite trend in control groups. There was no significant difference of the IL-6 concentrations in control groups’ colonic intestinal lavage fluid and plasma. It showed significantly lower concentrations of the IL-6 in DSS-treated groups’ miR-30c KO mice. The mRNA expression levels of relevant genes (IL-1β, IL-6, IL-10, IL-12, IL-23, and TNF-α) in the colonic mucosa were also measured. The result of mRNA expression in the colonic mucosa was shown in Fig. 2c. The mRNA expression trends of these cytokine were similar to those in plasma and the colonic intestinal lavage fluid.

### Low miR-30c expression accelerated histopathological damage in the colon of mice

**Figure 3:**
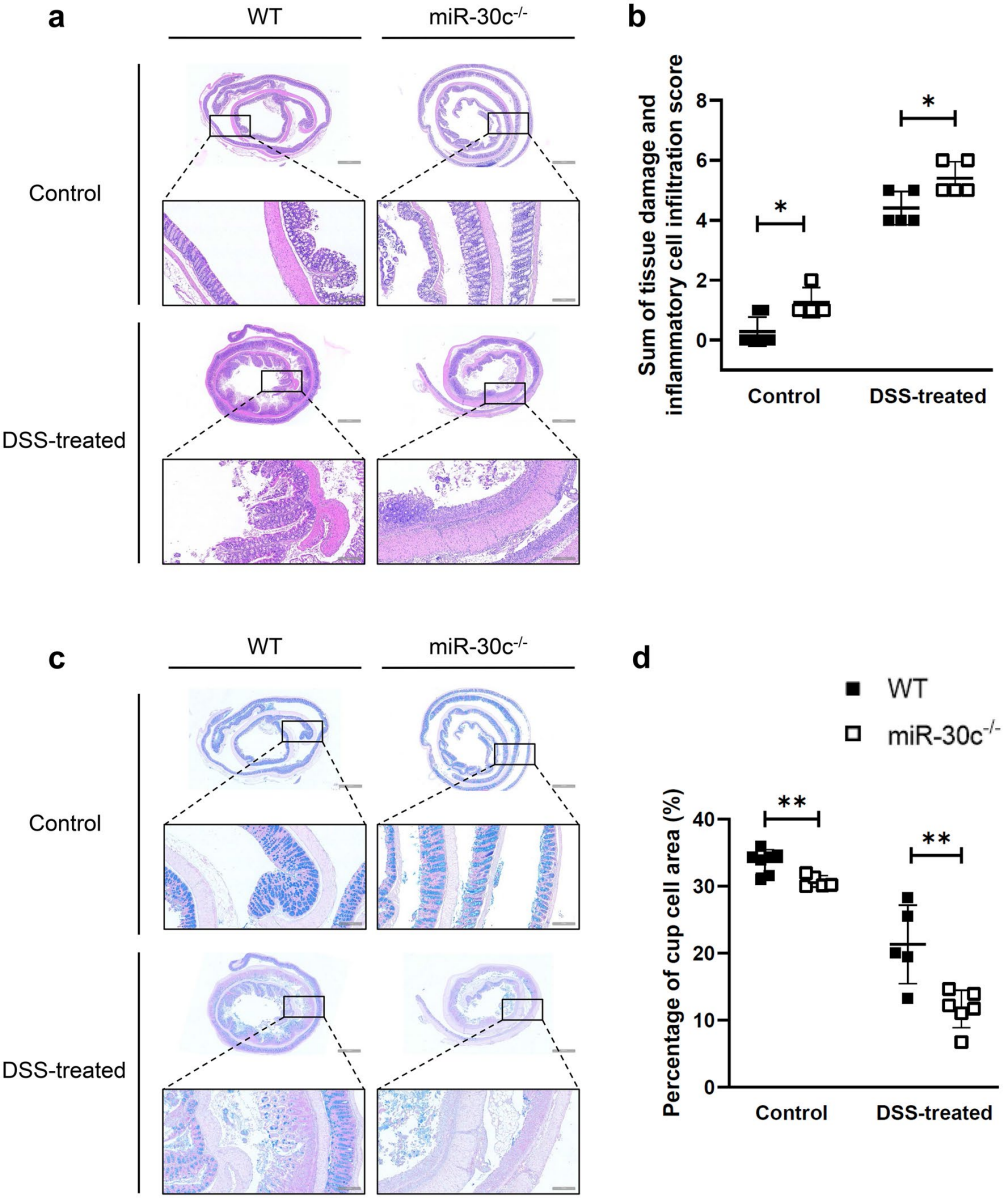
Low miR-30c expression accelerated histopathological damage in the colon of mice. (**a**) Representative histopathology images in the colon slides of different groups’ mice by H&E staining. (Low Mag: Scale bars, 1000 μm; High Mag: Scale bars, 200 μm) (**b**) Statistical analysis of the sums of tissue damage scoring and inflammatory cell infiltration scores in the colon slides of different groups’ mice and which were calculated as described in Materials and Methods (n = 4–6/group). (**c**) Representative images of gobletcells in the colon slides of different groups’ mice by AB-PAS staining. (Low Mag: Scale bars, 1000 μm; High Mag: Scale bars, 200 μm) (**d**) Statistical analysis of the percentages of occupied by gobletcells in the colon slides of different groups’ mice (n = 5–6/group). Data are presented as the means ± SD and were analyzed with Student’s t-test. (*P < 0.05; **P < 0.01).

In order to compare histopathological findings of colon injury, the colon samples of mice were taken to the shape of Swiss roll to make wax block and sectioned. Sections were stained with hematoxylin and eosin (HE) and scored. It was observed by HE staining that WT mice in the control group had normal crypt structure and normal arrangement of colonic mucosal epithelial cells. The colons of miR-30c KO mice had the pathohistological features of spontaneous mild colitis. There were slightly disorganized epithelial cell arrangements in the colonic mucosa and a little inflammatory cell infiltration in the lamina propria. Thus miR-30c KO mice had significantly higher histopathologic scores of colon of mice than WT mice in control groups. In the DSS-induced groups, there were irregular crypt structures in WT mice colons, but still mucosal epithelial cells were arranged. It showed the epithelium of miR-30c KO mice colon necrotic and detached; there were complete absence of crypt structures and disruption of glandular; the colonic mucosal layer and submucosa were heavily infiltrated with inflammatory cells, resulting in submucosal edema. It showed significantly higher scoring in colon histopathology scores of miR-30c KO mice in DSS-treated groups (Fig. 3a,b). Sections were stained with AB-PAS to determine the percentage of goblet cell area. In the control groups, it showed a few abnormal crypt structures in the mucosal epithelial layer of miR-30c KO mice colons, and the area of the mucin region was lower than that of WT mice. Thus, the area occupied by goblet cells was significantly lower than that of WT mice. In the DSS-treated groups, there were still a few mucins in some mucosal epithelial layers of the colons of WT mice. In contrast, there were nearly no mucin areas in the colon of miR-30c KO mice, which completely disappeared at the ulcers. As a result, it showed that the area occupied by goblet cell was significantly lower than that of WT mice by nearly double (Fig. 3c,d).

### The targeted regulation between VIP and miR-30c

**Figure 4:**
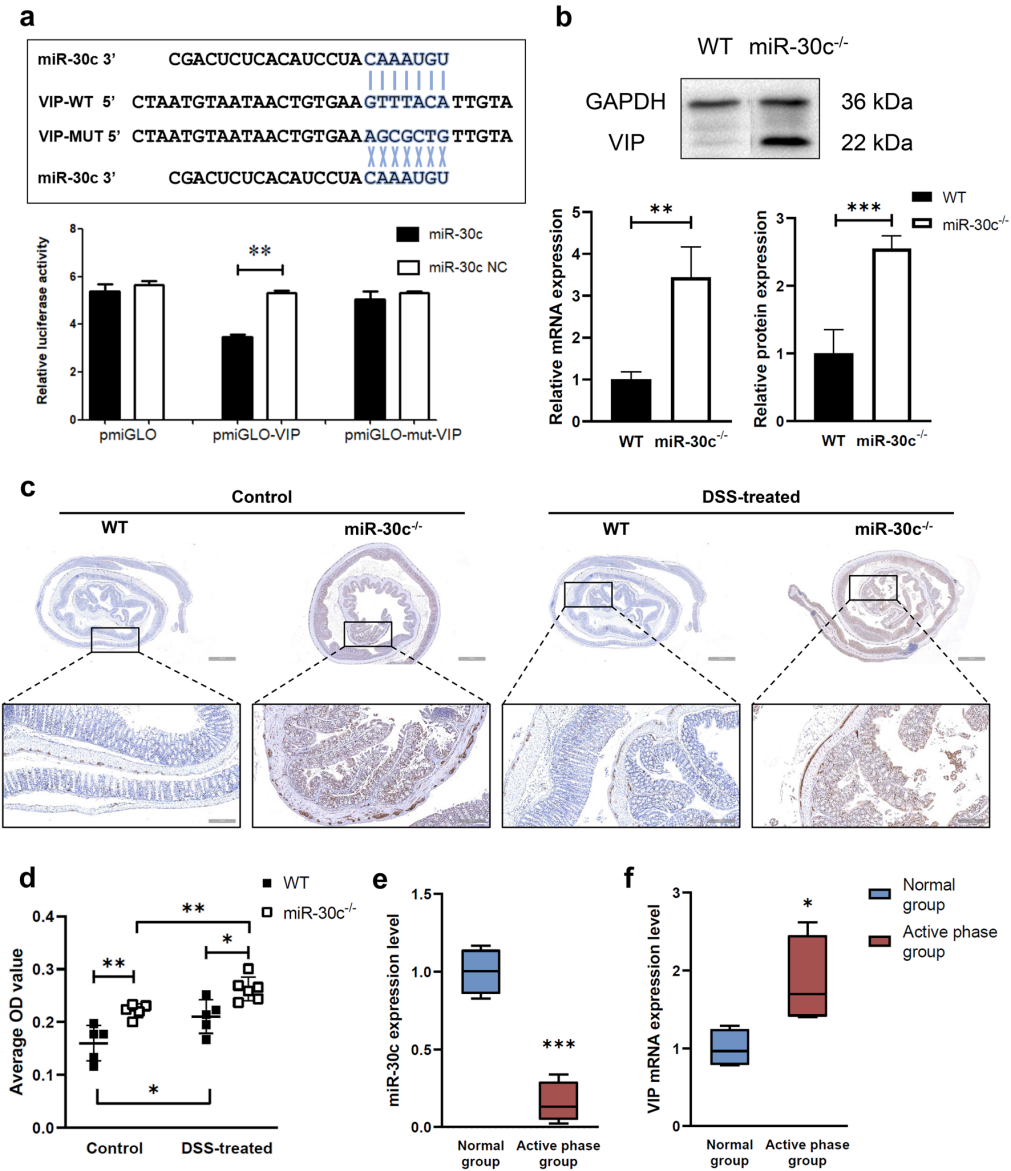
VIP is a direct target of miR-30c. (**a**) Wild type (WT) as well as mutated type (MUT) sequences of VIP for miR-30c target and dual luciferase report assay of VIP 3’-UTR wild type or MUT along with miR-30c. (**b**) The expression level of VIP was detected by qRT-PCR and Western Blot in the colon tissues of WT and miR-30c KO mice (n = 3). (**c**) Representative histopathology images in the colon slides of different groups’ mice by IHC staining for VIP. (Low Mag: Scale bars, 1000 μm; High Mag: Scale bars, 200 μm) (**d**) Statistical analysis of the average optical density values of positive signals in the colon slides of different groups’ mice (n = 5–6). (**e**) The expression level of miR-30c in the colon tissues of in the active ulcerative colitis patients and normal healthy control individuals (n = 4/group). (**f**) The expression level of VIP in the colon tissues of in the active ulcerative colitis patients and normal healthy control individuals (n = 4/group). Data are presented as the means ± SD and were analyzed with Student’s t-test. (*P < 0.05; **P < 0.01; ***P < 0.001).

Wild type (WT) VIP 3’-UTR (predicted to interact with miR-30c) cloned into a luciferase reporter vector was to demonstrate a direct interaction between VIP 3’-UTR and miR-30c. The luciferase intensity was reduced by approximately 30% in the 293 T cell line when cells were cotransfected with miR-30c mimic. The mutated (MT) version of VIP 3'-UTR was used as a control, and its 7 bases complementary to miR-30c were substituted. As expected, there was no change in luciferase activity (Fig. 4a). The expression level of VIP was detected by qRT-PCR and Western Blot in the colon tissues of WT mice and miR-30c KO mice to further validate that VIP is a direct target of miR-30c. It showed that the expression level of VIP in the colon of miR-30c KO mice was significantly higher than that of WT mice (Fig. 4b). The full-length blot image was included in Supplement Figure S1. Combined with the immunohistochemical results, the epithelial layer of the colon of mice had different degrees of damage (Fig. 4c). The expression level of VIP in the colon of miR-30c KO mice was significantly higher than that of WT mice by comparing the average OD value of the slices. After DSS induction, VIP expression was also slightly elevated in the colon of mice (Fig. 4d). As summarized, there is a targeted regulation between VIP and miR-30c and it plays a role in UC.

### Low expression levels of miR-30c in human colon tissues

To investigate the expression levels of miR-30c and VIP in human colon tissues, a total of 4 active ulcerative colitis patients (male; age range: 26–35 years; the Sutherland Index range: 8–11; the modified Baron score range: 3–4) and 4 normal controls (healthy individual; males; age range: 35–66 years) were included in this study. The expression levels of miR-30c in the colon tissues of the active ulcerative colitis patients were significantly lower than that in normal controls (Fig. 4e). However, the expression levels of VIP in the colons of the active ulcerative colitis patients was significantly higher than that in normal controls (Fig. 4f).

## Discussion

The results of this study showed that miR-30c KO mice with DSS-induced colitis showed more severe phenotypes: significantly higher disease activity indices, significant body weight loss, reduced length of the colon of mice, increased number of aberrant crypt structures, reduced mucus secretion, and significant differences in inflammatory factors. miR-30c is belonged to the the microRNA-30 family^20^. There was an indication that the microRNA-30 family in peripheral blood mononuclear cells of IBD patients could mediate IBD pathogenesis through adsorption by molecular sponges^21^.. It also has been reported that miR-30 family played an important role in the development of many diseases. In addition to affecting the consumption of the body by inflammation^22–24^, it also regulates epithelial mesenchymal transition in tumors^25–27^. Althoug, the miR-30c expression disorders play a role in activating and regulating various signaling pathways for tumorigenesis, invasion and metastasis in digestive organs^28–30^ the role of miR-30c in IBD, especially UC, has rarely been reported so far. In this study, DSS-induced colitis in mice was used to investigate the effect of low miR-30c expression on colon of mice. It was found that low expression of miR-30c exacerbated the symptoms of colitis and promoted the associated immune response in mice, resulting in more severe damage to the colon of mice.

It is crucial that the role of miRNAs in the gut is highly influential. There are various roles that miRNAs perform, ranging from intestinal epithelial barrier defects to dysregulated immune responses to flora imbalance. In addition, miRNAs can play multiple roles in regulating the dynamic balance of the gut. It has been shown that elevated levels of IL-1β mRNA and miR-200c expression in the intestinal tissues of UC patients and DSS-induced mice down-regulated the expression of occludin, thereby increased intestinal permeability^31^. It was similarly found that the concentration of IL-1β in mice was elevated and the colonic mucosa was severely damaged after we induced colitis in miR-30c KO mice with DSS. In addition, more and more laboratories are utilizing gene chip technology to screen for miRNAs that are differentially expressed in the UC and are trying to identify their target genes as objectives for further research^32^. Therefore, after finding VIP as one of the target genes of miR-30c, we validated VIP, which has demonstrated to control the development of ulcerative colitis^33^. It was reported that the expression of VIP was upregulated in plasma of IBD patients and in DSS-induced mice^19^. Similarly, whether miR-30c KO mice or not, after DSS-induced colitis, we detected upregulation of VIP expression in the colon of mice. Altogether our results suggest that targeted regulation of VIP by miR-30c exacerbates the pathogenesis of UC.

Although we have not investigated the detailed mechanisms of VIP in UC, notably, the role of VIP in the onset and progression of IBD remains controversial. The lower dose of VIP can increase the severity of colitis in a mouse model of Crohn's disease, found by Newman et al^34^. In contrast, John P et al. showed that the absence of VIP expression could induce colitis resistance^35^. It appears that VIP exercises a double-edged sword-like role. It was discovered by Abad et al. that low doses of VIP could reduce the severity of colitis, while high doses could worsen it^36^. In any case, our study showed that miR-30c, which targets and regulates VIP, plays an important role in the pathogenesis of UC. Therefore, the pathway designed by miR-30c expression and target genes may become a promising therapeutic target for the diagnosis, prognosis and treatment of colitis-associated cancers. We supposed that it’s worthy to further explore the mechanisms of miR-30c and VIP in the development of UC.

However, there are several limitations to this study. First, the sample size of human colon tissue was relatively small. More samples, are needed to identify the expressions of miR-30c and VIP among active ulcerative colitis patients and healthy control individuals. Also, it would be better to compare miR-30c and VIP expression levels of remission state patients to active ulcerative colitis patients, which would provide more information in the process of ulcerative colitis. Second, the study only focused on VIP, which was identified the target gene of miR-30c in this study. It is also important to investigat other miR-30c's downstream targets for a comprehensive understanding of its involvement in ulcerative colitis. Third, this study did not assess the specificity and sensitivity of the identified miR-30c and VIP in distinguishing ulcerative colitis from other inflammatory bowel disease diseases.

In the summary, this study identified that the absence of miR-30c might promote DSS-induced colitis, and the targe-regulatory effect of miR-30c on VIP might play an important role in the development of colitis.

## Methods

### Active ulcerative colitis patients and normal healthy control individuals

The colon tissue samples of 4 active ulcerative colitis patients and 4 normal healthy controls were used to analyze miR-30c and VIP expression levels in this study, which were provided by the First Affiliated Hospital of Bengbu Medical College. The patients were included into this study according to the score of Sutherland Index (to describe the disease activity)^37^ and the modified Baron score (to represent an endoscopic classification)^38^. This study was approved by the Ethics Committee of Bengbu Medical College (No.2023LK401) and was conducted in accordance with the ethical guidelines of the Declaration of Helsinki. All participants in this study were over the age of 18, and informed consent was obtained from all subjects. We are committed to protecting the privacy and personal information of our participants throughout the research process and ensuring that all procedures performed in the study adhere to the applicable ethical standards.

### Animals

The miR-30c gene knockout mice (miR-30c^-/-^, referred to as KO) used in this study were utilized by using CRISPR/cas9-mediated genome engineering technology (Shanghai Model Organisms Center, China). The target sequences of two sgRNAs were 5'-AAGTGTCCATGACAGTGTCA-3' and 5'-ACTAGACTTAGATGCTCTGC-3', which targeted the transcript of mmu-mir-30c-1 (ENSMUST00000083556.3). miR-30c KO mice were further validated by qRT-PCR test. The mice were fed and watered liberally under specific pathogen free (SPF) conditions, standardized temperature conditions (21–22 °C) and illumination (12 h light/12 h dark). All animal experimental protocols were approved by the Institutional Animal Care and Use Committee of Bengbu Medical College (No.2022LDK140). And methods were carried out in accordance with relevant guidelines and regulations. This study was carried out in compliance with the ARRIVE guidelines.

### DSS-induced colitis in mice

To induce experimental colitis, it was established by providing the mice with 2.5% (wt/vol) DSS (MP Biomedicals, Santa Ana, CA, USA; molecular weight of 36,000–50,000 Da; CAS Number: 9011-18-1) dissolved in drinking water for 7 days. There were 4 groups (6 mice per group): WT and miR-30c KO mice untreated group (normal water without DSS); WT and miR-30c KO mice DSS treated group (water containing 2.5% DSS). Daily weight, diarrhea, and bleeding scores were recorded. The weight loss percentage was scored as: grade 0, none; grade 1, 1 to 5%; grade 2, 5 to 10%; grade 3, 10 to 15%; grade 4, > 15%. Fecal bleeding was scored by Occult blood test kit (O-toluidine method, Yuanye Bio, China) and observation as grade 0, no bleeding and tested negative; grade 1, tested weakly positive; grade 2, some bleeding with tested positive; grade 3, gross bleeding; grade 4, blood filling the whole colon. The score for stool consistency was: grade 0, normal stool; grade 1, slightly loose stool; grade 2, loose stools; grade 3, watery stool; grade 4, severe diarrhea (according to Cooper and colleagues, with modifications). Disease Activity Index DAI = (Decreased Body Mass Score + Blood in Stool Score + Fecal Characteristics Score).

### The levels of IL-1β, IL-6, IL-10, IL-12, IL-23, and TNF-α in plasma and colonic lavage fluid

The plasma were obtained by orbital blood sampling and the colons were taken and cleaned with PBS (phosphate buffered saline) solution. The levels of inflammatory factors (IL-1β, IL-6, IL-10, IL-12, IL-23, and TNF-α) in plasma and colonic lavage fluid were measured using ELISA (Enzyme-Linked Immunosorbent Assay) kits (Jingmei Bio, China) according to the manufacturer's instructions.

### Analysis and scoring of histopathology

After the fresh mice colon tissues were rolled up and fixed with 10% formaldehyde, the tissues were dehydrated, transparent, and were dipped in wax to make paraffin-embedded tissues. 10 µm-sections of tissues were taken for deparaffinization and rehydration. Slides were blocked with arborvitae after hematoxylin and eosin (H&E) staining, Alcian blue and Periodic acid Schiff (AB-PAS) staining. They were observed by microscopy (Olympus, Japan) and evaluated using Image J software. The sums of tissue damage scoring and inflammatory cell infiltration scoring were used for statistical analysis, both of which were scored on the following scale: grade 0, no colon mucosal damage, and few scattered inflammatory cells in the lamina propria of the colonic mucosa; grade1, colonic epithelial cells are damaged,and massive invasion of inflammatory cells into the lamina propria of the colonic mucosa; grade 2, colonic mucosal damage or with focal ulcers, and widespread inflammatory cells with extension into the colonic submucosa; grade 3, damage to the colonic mucosa that spreads to deeper colonic wall structures,and massive invasion of inflammatory cells into the colonic submucosa. The goblet cells were also counted on AB-PAS staining results.

### RNA extraction and quantitative real‑time polymerase chain reaction (qRT‑PCR) assay

Total RNA was isolated using the MiFure Cell/Tissue miRMA Kit (Vazyme, China). miR-30c expression was reverse transcribed and detected according to All-in-One miRNA qRT-PCR Detection Kit 2.0 (GeneCopoeia, China) and U6 was used as endogenous controls. The expression levels of VIP, IL-1β, IL-6, IL-10, IL-12, IL-23, and TNF-α was measured using GAPDH as the reference gene by qRT-PCR (Applied Biosystems™ 7500, USA). Relative gene expression level was calculated by 2^−ΔΔCt^ relative quantitative method. The primer sequences were listed in the Supplement Table S1.

### Cell culture and plasmid construct for luciferase reporter assay

The 293 T cell line (KeyGEN BioTECH, China) was cultured in DMEM (GIBCO, USA). 10% fetal bovine serum (FBS) (ExCell Biology, USA) in the incubator at 37 °C and 5% CO_2_. The VIP 3'-UTR sequence and the mutated VIP 3'-UTR sequence were designed and synthesized in order to verify the targeting relationship between miR-30c and VIP. The synthesized fragments of the two target genes were cloned into pmirGLO dual luciferase reporter gene vectors to construct the VIP 3'-UTR dual luciferase reporter gene wild-type vector (pmirGLO-VIP) and its mutant vector (pmirGLO-mut-VIP), respectively. Recombinant vectors were identified by PCR electrophoresis and gene sequencing. The two recombinant vector plasmids were co-transfected with miR-30c mimics (mimics) or miR-30c Negative Control (NC) in 293 T cells using the transfection reagent lipofectamineTM2000, respectively. The activity of luciferase was detected using the Dual-Luciferase Assay System Kit (Promega, Madison, USA) by Multi-function Enzyme Labeler (Molecular Devices M3, USA).

### Antibodies and Western Blot analysis

The terminal colon proteins of mice were extracted by RIPA Lysis Buffer kit (Beyotime, China). The extracted proteins were quantified by BCA Protein Assay Kit (Epizyme, China), and the protein expression was analyzed by Western blotting assay. Membranes were incubated overnight at 4 °C with the primary antibodies VIP (DF6627, Affinity Biosciences, China) and GAPDH (AF7021, Affinity Biosciences, China) mixed by primary antibody diluent (Epizyme, China) and incubated with Goat Anti-Rabbit IgG (H + L) HRP (S0001, Affinity Biosciences, China) subsequently. Immunodetected proteins were visualized using YosiSuper West Pico PLUS Chemiluminescent Substrate Kit (Yoshi Bio, China). The protein expression was quantified using the ImageJ software (version 1.53 s; ImageJ can be downloaded from https://imagej.net/ij/download.html).

### Immunohistochemical analysis

The slides were incubated with diluted primary antibody at 4 °C overnight followed by secondary antibody incubation. Staining was performed as described in the operating manual of DAB Horseradish Peroxidase Color Development Kit (Solarbio, China). The antibodies were the same as those used in the WB analysis. And the average optical density values of positive signals were analyzed using the ImageJ software.

### Statistical analysis

Data were presented as means ± standard deviation (SD) using R software version 4.0.3. The experimental data were analyzed using an unpaired two-tailed t-test. *P* values < 0.05 were considered statistically significant.

### Supplementary Information

Supplementary Information.

## Data Availability

All data generated or analysed during this study are included in this published article and its supplementary information files.
