# Supplementary material for: miR-30c affects the pathogenesis of ulcerative colitis by regulating target gene VIP
Source: Sci Rep. 2024 Feb 12;14:3472. doi: 10.1038/s41598-024-54092-y (PMC10859366; doi:10.1038/s41598-024-54092-y)

Title page

miR-30c affects the pathogenesis of ulcerative colitis by regulating target gene VIP

Xiang Dong[1,2], Yuling Zhan[1,2], Minghui Yang[2,3], Suwan Li[1,2], Hailun Zheng[4], Yu Gao[1,5,6*]

[1] School of Life Science, Bengbu Medical College, Bengbu, China

[2] Bengbu Medical College Key Laboratory of Cancer Research and Clinical Laboratory Diagnosis, Bengbu Medical College, Bengbu, China

[3] School of Basic Courses, Bengbu Medical College, Bengbu, China

[4] Department of Gastroenterology, the First Affiliated Hospital of Bengbu Medical College, Bengbu, China

[5] Anhui Province Key Laboratory of Translational Cancer Research, Bengbu Medical College, Bengbu, China

[6] Laboratory Animal Center, Bengbu Medical College, Bengbu, China

* **Corresponding Author:** Yu Gao, School of Life Science, Laboratory Animal Center, Bengbu Medical College, No. 2600 Donghai Road, Bengbu, 233030, China. Tel: +86-19105521190. Fax: +86-552-3175396. Email: gaoyu@bbmc.edu.cn

Supplemental Table S1. Primer sequences for qRT-PCR

Gene name	Primer sequence 5'-3'
U6	Forward Primer CTCGCTTCGGCAGCACA
	Reverse Primer AACGCTTCACGAATTTGCGT
miR-30c-5p	Forward Primer TGTAAACATCCTACACTCTCAGCAA
	Reverse Primer GCTGTCAACGATACGCTACGTAACG
mGAPDH	Forward Primer AGGTCGGTGTGAACGGATTTG
	Reverse Primer TGTAGACCATGTAGTTGAGGTCA
mVIP	Forward Primer AGTGTGCTGTTCTCTCAGTCG
	Reverse Primer GCCATTTTCTGCTAAGGGATTCT
hGAPDH	Forward Primer GGAGCGAGATCCCTCCAAAAT
	Reverse Primer GGCTGTTGTCATACTTCTCATGG
hVIP	Forward Primer GACACCAGAAATAAGGCCCAG
	Reverse Primer GTCACCCAACCTGAGAGCAG
mIL-1β	Forward Primer GCAACTGTTCCTGAACTCAACT
	Reverse Primer ATCTTTTGGGGTCCGTCAACT
mIL-6	Forward Primer TAGTCCTTCCTACCCCAATTTCC
	Reverse Primer TTGGTCCTTAGCCACTCCTTC
mIL-10	Forward Primer GCTCTTACTGACTGGCATGAG
	Reverse Primer CGCAGCTCTAGGAGCATGTG
mIL-12α	Forward Primer CTGTGCCTTGGTAGCATCTATG
	Reverse Primer GCAGAGTCTCGCCATTATGATTC
mIL-23α	Forward Primer ATGCTGGATTGCAGAGCAGTA
	Reverse Primer ACGGGGCACATTATTTTTAGTCT
mTNFα	Forward Primer CCCTCACACTCAGATCATCTTCT
	Reverse Primer GCTACGACGTGGGCTACAG

Supplement Figure S1. The full-length blot image for VIP and GAPDH.

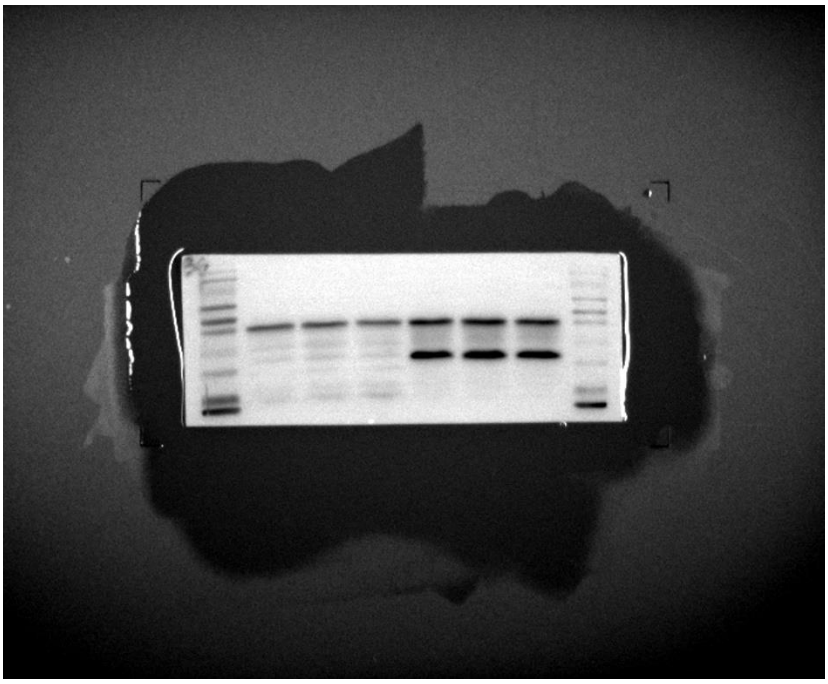

Supplement: Supplementary file 1 — Supplementary Information. [file 41598_2024_54092_MOESM1_ESM.pdf]
